# Inhibitory Effect of Ursodeoxycholic Acid on *Clostridium difficile* Germination Is Insufficient to Prevent Colitis: A Study in Hamsters and Humans

**DOI:** 10.3389/fmicb.2018.02849

**Published:** 2018-11-22

**Authors:** Lola-Jade Palmieri, Dominique Rainteau, Harry Sokol, Laurent Beaugerie, Marie Dior, Benoit Coffin, Lydie Humbert, Thibaut Eguether, André Bado, Sandra Hoys, Claire Janoir, Henri Duboc

**Affiliations:** ^1^ERL INSERM U1157/UMR7203, PM2, Assistance Publique-Hôpitaux de Paris (AP-HP), Faculté de Médecine Saint-Antoine, Sorbonne Université, Paris, France; ^2^EA4043 Unité Bactéries Pathogènes et Santé (UBaPS), Université Paris-Sud, Université Paris-Saclay, Châtenay-Malabry, France; ^3^INSERM U1149, Centre de Recherche sur l’Inflammation, Faculté de Médecine Paris Diderot, Université Paris Diderot, Paris, France; ^4^Department of Gastroenterology and Nutrition, Saint Antoine Hospital, Assistance Publique-Hôpitaux de Paris (AP-HP), Paris, France; ^5^Department of Gastroenterology, Louis Mourier Hospital, Assistance Publique-Hôpitaux de Paris (AP-HP), Paris, France

**Keywords:** *Clostridium difficile*, ursodeoxycholic acid, bile acids, growth, germination, inflammatory bowel disease, primary sclerosing cholangitis

## Abstract

**Introduction:** Bile acids (BA) influence germination and growth of *Clostridium difficile*. Ursodeoxycholic acid (UDCA), a BA minor in human, used for cholestatic liver diseases, inhibits germination and growth of *C. difficile in vitro*, but was never tested *in vivo* with an infectious challenge versus control. We hypothesized that UDCA could prevent CDI. We evaluated the effects of UDCA on *C. difficile in vitro* and in hamsters, with pharmacokinetics study and with an infectious challenge. Then, we studied CDI incidence in UDCA–treated patients.

**Methods:** We evaluated germination and growth of *C. difficile*, with 0.01, 0.05, and 0.1% UDCA. We analyzed fecal BA of hamsters receiving antibiotics and UDCA (50 mg/kg/day), antibiotics, or UDCA alone. Then, we challenged with spores of *C. difficile* at D6 hamsters treated with UDCA (50 mg/kg/day) from D1 to D13, versus control. In human, we analyzed the database of a cohort on CDI in acute flares of inflammatory bowel disease (IBD). As PSC-IBD patients were under UDCA treatment, we compared PSC-IBD patients to IBD patients without PSC.

**Results:** *In vitro*, UDCA inhibited germination and growth of *C. difficile* at 0.05 and 0.1%, competing with 0.1% TCA (with 0.1%: 0.05% ± 0.05% colony forming unit versus 100% ± 0%, *P* < 0.0001). In hamsters, UDCA reached high levels only when administered with antibiotics (43.5% UDCA at D5). Without antibiotics, UDCA was in small amount in feces (max. 4.28%), probably because of UDCA transformation into LCA by gut microbiota. During infectious challenge, mortality was similar in animals treated or not with UDCA (62.5%, *n* = 5/8, *P* = 0.78). UDCA percentage was high, similar and with the same kinetics in dead and surviving hamsters. However, dead hamsters had a higher ratio of primary over secondary BA compared to surviving hamsters. 9% (*n* = 41/404) of IBD patients without PSC had a CDI, versus 25% (*n* = 4/12) of PSC-IBD patients treated with UDCA.

**Conclusion:** We confirmed the inhibitory effect of UDCA on growth and germination of *C. difficile in vitro*, with 0.05 or 0.1% UDCA. However, in our hamster model, UDCA was inefficient to prevent CDI, despite high levels of UDCA in feces. Patients with PSC-IBD treated with UDCA did not have less CDI than IBD patients.

## Introduction

*Clostridium difficile* infection (CDI) is a severe digestive infection due to a Gram-positive anaerobic and sporulating bacterium, mainly triggered by antibiotherapy. CDI is a public health threat and a financial burden. In 2011, half a million cases were estimated in the United States, leading to 29,000 deaths (Lessa et al., 2015). One of the man concerns is the frequency of recurrence, which is associated with repetitive hospitalizations or longer stays (Aitken et al., 2014; Lübbert et al., 2016; Sheitoyan-Pesant et al., 2016). Antibiotics are recommended as treatment for first recurrence. European guidelines indicate fecal microbiota transplant for a second recurrence (Debast et al., 2014) and American guidelines (Surawicz et al., 2013) for the third. Fecal microbiota transplant is an efficient, recent but costly new treatment (van Nood et al., 2013), with limited visibility on potential long-term risks, especially for metabolism (Ridaura et al., 2013; Alang and Kelly, 2015) and immunity (Brandt et al., 2012). Contamination by *C. difficile* occurs by environmental spore ingestion. Spores become pathogenic when they germinate into vegetative cells, followed by the growth of the vegetative cells able to produce the toxins responsible for enteric lesions. The mechanism of germination of *C. difficile* spores depends on Bile Acids (BA): taurocholic acid (TCA) efficiently triggers germination *in vitro* (Wilson et al., 1982). In human and hamster the primary BA, cholic acid (CA) and chenodeoxycholic acid (CDCA), are produced by the liver conjugated to taurine or glycine and excreted in the bile. CA and CDCA are transformed into secondary BA in the gut by microbiota, respectively, in deoxycholic acid (DCA) and lithocholic acid (LCA) (Ridlon et al., 2006). A germination-specific protease, CspC, has been identified as a *C. difficile* germinant receptor for BA (Francis et al., 2013a; Bhattacharjee et al., 2016). CA triggers germination, but, on the contrary, some BA are strong inhibitors of germination or growth *in vitro*. LCA and CDCA inhibit both germination and growth (Sorg and Sonenshein, 2010; Theriot et al., 2016; Weingarden et al., 2016), while DCA triggers germination (Sorg and Sonenshein, 2008; Theriot et al., 2016) but inhibits growth (Sorg and Sonenshein, 2008; Buffie et al., 2015; Theriot et al., 2016). In human colon BA pool, there are 10% of primary BA and more than 90% of secondary BA. This composition is theoretically unfavorable for germination and subsequent growth of *C. difficile*. By disrupting microbiota, antibiotherapy leads to a decrease in secondary BA, whereas primary BA increase – including CA that triggers germination. It has been proved that fecal microbiota transplantation restores low levels of CA, probably by restoring a functional BA transformation activity (Weingarden et al., 2014).

Ursodeoxycholic acid (UDCA) is quantitatively minor in humans (less than 2% of the BA pool). This tertiary BA, produced from CDCA and LCA, is widely used for the treatment of cholestatic liver diseases: in primary biliary cholangitis at 13–15 mg/kg/day, in primary sclerosing cholangitis (PSC) at 15–20 mg/kg/day, and up to 30 mg/kg/day in some genetic chronic cholestasis (European Association for the Study of the Liver [EASL], 2009). UDCA inhibits germination and growth *in vitro* (Weingarden et al., 2015; Theriot et al., 2016). *In vivo*, one study tested UDCA for a patient with a colectomy for inflammatory bowel disease (IBD) and a recurrent *C. difficile* pouchitis: it was successful without long-term relapse (Weingarden et al., 2015). UDCA would present interesting advantages: it has been part of the human pharmacopeia for years, its side effects are known and minor (diarrhea) and its price is low.

We assumed that UDCA could be used as a preventive treatment for CDI. First, we aimed to confirm UDCA ability to inhibit germination and growth of *C. difficile in vitro*. Then, we conducted pharmacokinetic tests and we tested the ability of UDCA to prevent CDI in hamsters. Finally, we analyzed data from a cohort studying the CDI in acute flares of IBD (Sokol et al., 2017), by comparing two subgroups: patients with PSC (thus treated with UDCA) and patients without PSC (thus untreated).

## Materials and Methods

### *In vitro* Studies

#### Reactives, Strains, and Growth Conditions

*C. difficile* strains (VPI10463, R20291, and 630Δ*erm)*, conserved at −80°C, were grown in BHIS [37 g/L brain heart infusion (Becton Dickinson) supplemented with yeast extract (5 g/L) and L-cysteine (1 g/L)] at 37°C under anaerobic conditions (90% N2, 5% CO_2_ and 5% H2), in an anaerobic chamber (Jacomex). UDCA was supplied from Mayoli and TCA from Sigma.

#### Preparation of *C. difficile* Spores

Sporulation of *C. difficile* was induced on Sporulation Medium (SMC) (Bacto-Peptone 90 g/L, Protease Peptone 5 g/L, NH_4_SO_4_ 1 g/L, Tris base 1.5 g/L, and agar 15 g/L) for *C. difficile* 630Δ*erm*, and on BHIS for *C. difficile* VPI 10463; 200 μL of overnight suspension of *C. difficile* were plated on agars, then incubated anaerobically for 5–7 days at 37°C. Plates were then scraped up with 3 mL of PBS: pooled suspension was washed three times with 40 mL of sterile water. The last spore pellet was resuspended in 10 mL of sterile water and stored at + 4°C for 7 days. This spore preparation was used for all *in vitro* experiment (except germination assay) and infection of hamsters. For germination experiments, spore preparations of 630Δ*erm* strain were further purified by density gradient centrifugation. The suspension was again washed three times (as above), and the pellet was resuspended in 1 mL of sterile water and centrifuged at 6000 rpm for 2 min. The pellet was mixed with 500 μL of 30% Radioselectan (Schering SAS laboratory) and the suspension was layered on 1 mL of 50% Radioselectan. The gradient was then centrifuged for 15 min at 15,000 rpm. The spore pellet was washed three times in 500 μL of sterile water to remove all traces of Radioselectan. Spores purity (>95%) was checked under optical microscopy.

#### *In vitro* Studies: Effect of UDCA on Germination and Growth of *C. difficile*

In order to determine the minimum concentration of UDCA inhibiting *C. difficile* growth, vegetative cells of strains VPI10463, R20291, and 630Δ*erm* were inoculated into Brain Heart Infusion-Supplemented (BHIS) medium to an optical density at 600 nm (OD_600_) of 0.05 and were grown anaerobically at 37°C until an OD_600_ between 0.4 and 0.6. Suspensions were diluted at 1:100. UDCA – dissolved in 100% ethanol – was added in order to obtain final concentrations of 0.001, 0.01, or 0.1%. All suspensions contained 2% ethanol. Suspensions were grown anaerobically at 37°C, and minimum inhibiting concentration and OD_600_ were determined 24 h after inoculation. Impact of UDCA on the growth kinetics was also determined. Vegetative cells were inoculated into BHIS containing UDCA 0.01, 0.05, or 0.1% to an OD_600_ of 0.1 and anaerobic growth was monitored by measurements of OD_600_ each hour during 10 h and at 24 h after inoculation. To determine the effect of UDCA on germination, purified spores (strain 630Δerm) were heat activated for 30 min at 65°C, then diluted to an OD_600_ of 0.7–1 in BHIS containing TCA 0.1% alone, or TCA 0.1% plus UDCA 0.01, 0.05, or 0.1%. OD_600_ was determined every minute for 20 min. The ratios of the OD_600_ at various time points to the OD_600_ at time zero were plotted against time. Finally, we analyzed the effect of UDCA on germination and growth in competition with TCA. BHI plates containing TCA 0.1% with different concentrations of UDCA (0.01, 0.05, 0.1%) and two controls (UDCA 0.1%, TCA 0.1%) were inoculated with 100 μL of spores. The number of colonies forming unit (CFU) was counted visually after 48 h of growth. We considered plates with 0.1% TCA as the control group and the number of CFU recovered on these control plates as 100%.

### *In vivo* Studies

#### Ethics Statement

Animals’ experiments were conducted according to the European Union guidelines for the handling of laboratory animals and followed the 3-R rules. Central Animal Care Facilities (agreement 92-019-01) and Use Committee of University Paris-Sud approved the protocol (number 9109/2017030215219770). For infectious experiment, hamsters were assessed twice daily by a clinical score. In case of pre-mortem animal suffering score, hamsters were euthanized with pentobarbital [150 mg/kg, intraperitoneally (IP)].

#### Animals and Housing

Golden Syrian female hamsters were 8 weeks old (75–100 g, Janvier LABS, Le Genest-Saint-Isle). They were taken care of by the central animal facility of the Faculty (AnimEx platform) and were housed individually in isolator cages fitted with covers holding disposable polyester air filters. Bedding, cages, wire lids and filter covers were autoclaved before use, as food and water (given *ad libitum*).

#### Pharmacokinetics Experiment

Hamsters were divided into 3 groups of 6. Antibiotherapy was administered to simulate the conditions of the infectious challenge: Group A received antibiotherapy [clindamycin 50 mg/kg IP at day 1 (D1), gentamicin 5 mg/kg/day twice daily, orally, D1–D5] and UDCA (50 mg/kg/day, D1–D7). Group B received only antibiotherapy and group C only UDCA. We administered UDCA by gavage in 100% porcine gelatin capsules (Torpac Rodent, Fairfield, NJ, United States). The stools were sampled daily between 4 and 7 h after their emission from D0 to D8, and regularly until D46, to determine fecal BA composition.

#### Measurement of BA in the Digestive Tract, Entero-Hepatic, and Systemic Circulation

Three hamsters received UDCA and antibiotherapy, according to the same modalities, but only from D1 to D3. Three other hamsters received only UDCA from D1 to D3. Hamsters were sacrificed on D4. Gut limbs (duodenum, jejunum, terminal ileum) were separated in 10 cm segments, and content was flushed (1 mL of sterile PBS). Stools were sampled directly from the colon. Portal and cardiac blood were also collected.

#### Infectious Challenge

Hamsters were divided into 2 groups of 8. Group A was treated with UDCA (50 mg/kg/day, gelatin capsules, D1–D13), antibiotics (clindamycin 50 mg/kg/day IP, on D1 and gentamicin 5 mg/kg/day twice a day, by gavage, from D1 to D5) and was challenged with 10^4^ spores of VPI10463 at D6 by gavage. Group B, control group, received only antibiotics and was challenged with *C. difficile* spores, according to the same modalities. Stools were recovered between 4 and 7 h after their emission, and counts of spores and vegetative forms were performed immediately, by plating of *ad hoc* dilutions (Péchiné et al., 2013) either on selective agar plates (CLO, Biomérieux, for vegetative forms) or Columbia cysteine agar plates enriched in horse blood (3%), taurocholate (0.1%) and supplement (Oxoid, Dardilly, France, for spores and vegetative forms). All manipulations (dilution and plating) were performed in an anaerobic chamber, and plates were incubated anaerobically at 37°C for 24–48 h before colonies enumeration.

#### BA Analyses

BA were prepared and then separated by High Pressure Liquid Chromatography coupled to tandem Mass Spectrometry (HPLC-MS/MS), as previously described (Humbert et al., 2012). Quantitation of all BA species was based on a standardized range. The list of the 28 BA species measured in feces is reported in Supplementary Table 1. Results were reported in percentage of total BA, for each specific BA. When studying the primary/secondary BA ratio, we used the sum of CA and CDCA divided by the sum of LCA and DCA.

### CDI in IBD Patients With or Without UDCA

We used the database of a cohort studying CDI in acute flares of IBD (Sokol et al., 2017). All consecutive patients hospitalized for a flare in Saint-Antoine Hospital (Paris, France) from September 2012 to May 2014 were prospectively tested for CDI, defined by a clinical picture compatible and a microbiological evidence of toxin-producing *C. difficile* in stools. As PSC-IBD patients were under UDCA treatment, we compared PSC-IBD patients to IBD patients without PSC, thus untreated.

### Statistical Analysis

Data were reported as means ± standard error of the mean (SEM). OD_600_ and CFU were compared with one-way ANOVA; growth, germination and BA curves with two-way ANOVA. BA between surviving and dead hamsters were compared with Student *t*-test. Hamsters’ survival was evaluated with Kaplan–Meier method and compared with log-rank test. Statistical tests were performed using Prism version 6.00d (GraphPad Software, La Jolla, CA, United States). Statistical significance was set at a *P-*value of < 0.05.

## Results

### UDCA Strongly Inhibited *C. difficile* Germination and Growth *in vitro*

**FIGURE 1:**
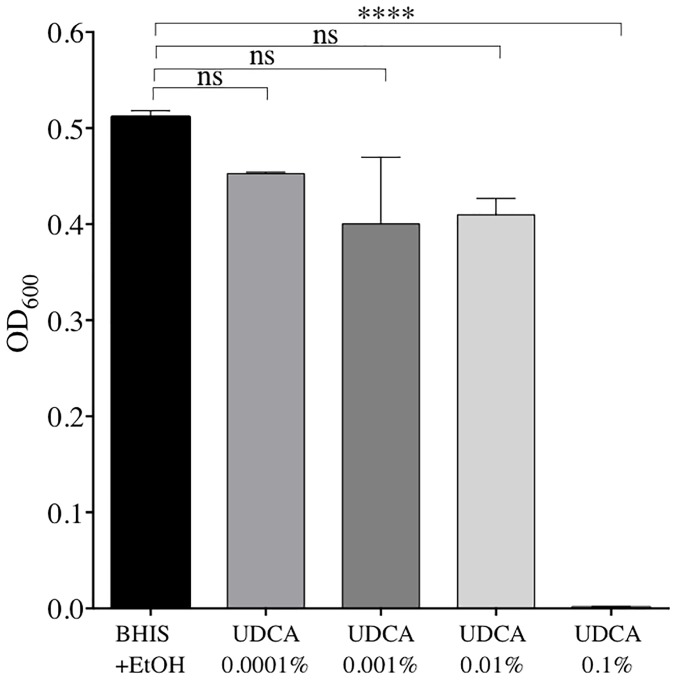
Optical densities (OD) after 24 h of growth of *C. difficile* strain VPI 10463 in Brain Heart Infusion Supplemented (BHIS) + ethanol (EtOH) 2% + ursodeoxycholic acid (UDCA): 0.0001, 0.001, 0.01, or 0.1%, and in BHIS + EtOH 2% alone. Data represent the standard error of the mean (SEM) from triplicate. A significant decrease in OD was reached with UDCA 0.1% compared to BHIS+EtOH (one-way ANOVA, ^∗∗∗∗^*P* < 0.0001).

The minimum concentration of UDCA to inhibit growth of vegetative forms of *C. difficile* VPI10463 was 0.1% UDCA. We confirmed this by measuring OD_600_ at 24 h (Figure 1). At 24 h, OD_600_ was strongly decreased with 0.1% UDCA, compared to control (0.001 ± 0.001 versus 0.511 ± 0.007, *P* ≤ 0.0001).

Second, we analyzed the effect of the inhibitory and sub-inhibitory concentrations of UDCA on growth kinetics of three strains of *C. difficile*. UDCA impacted the growth kinetics of vegetative forms with a dose-dependent effect. However, the strains did not behave exactly in the same manner. For the strain VPI10463, growth was significantly slowed down with UDCA at 0.1% (Supplementary Figure 1A). The 630Δ*erm* and R20291 strains tested were slightly more sensitive to UDCA. Their growth were significantly slowed down with UDCA 0.1% (*P* < 0.0001) but also with UDCA 0.05% (*P* < 0.0001, Supplementary Figures 1B,C).

Then, we determined the inhibitor effect of UDCA on the germination induced by TCA. Germination curves were performed with one sample of purified spores of the 630Δ*erm* strain. We observed that 0.1 and 0.05% UDCA seemed to inhibit the germination (Supplementary Figure 2). OD (t40)/OD (t0) was 94.6% with 0.1% UDCA, 94.4% with 0.05% UDCA, versus 82% in the control group without UDCA.

**FIGURE 2:**
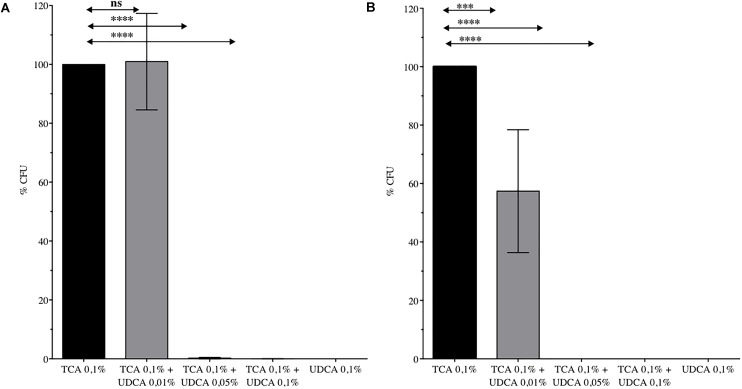
Percentage of Colony Forming Unit (CFU) issued from spores of *C. difficile* VPI 10463 **(A)** or 630 Δ*erm* **(B)** plated on Brain Heart Infusion – Agar with ursodeoxycholic acid (UDCA) 0.01, 0.05, or 0.1%. 100% CFU corresponds to CFU from spores of *C. difficile* plated on BHIS with taurocholate (TCA) 0.1%. Data represent the standard error of the mean (SEM) from triplicate. One-way ANOVA was performed. ns, non significant, ^∗∗∗∗^*P* < 0.0001, ^∗∗∗^*P* < 0.001.

Finally, we analyzed the global effect of UDCA (on germination and growth) by determining the number of CFU recovered from a spore preparation on plates containing 0.1% TCA, with or without UDCA. Inhibition of germination and growth was complete, for the strain VPI10463 with 0.05% (CFU ± SEM: 0.24% ± 0.24% versus 100% ± 0%, *P* < 0.0001) and 0.1% (0.05% ± 0.05% versus 100% ± 0%, *P* < 0.0001) of UDCA (Figure 2A). For 630Δ*erm*, a significant decrease of CFU was observed with 0.01, 0.05, and 0.1% UDCA (Figure 2B).

### *In vivo* Experiments

#### Pharmacokinetics Experiment – A High Percentage of UDCA in the Fecal Pool Was Reached Only When Hamsters Received Antibiotics Simultaneously

**FIGURE 3:**
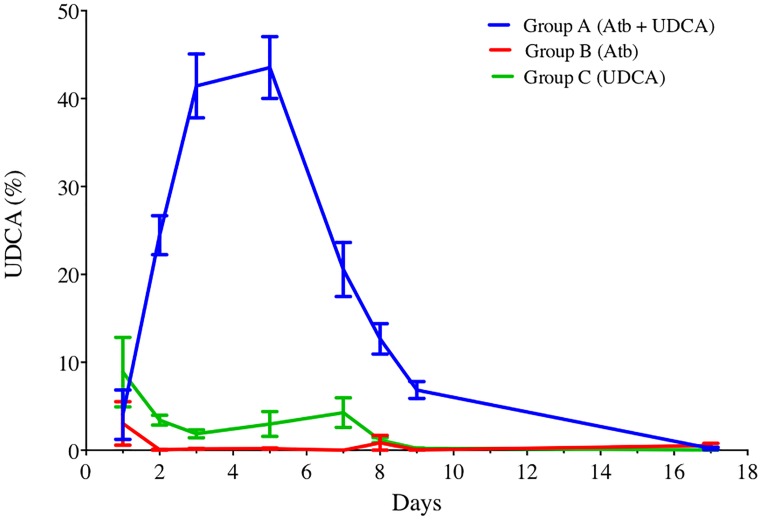
UDCA percentages in feces of hamsters over time, in groups A, B, and C. Antibiotics were administrated from D1 to D5 and UDCA was administrated from D1 to D8. Data represent the standard error of the mean (SEM) from 6 hamsters. *P* < 0.0001 with two-way ANOVA. Atb, antibiotics; UDCA, ursodeoxycholic acid.

UDCA gavage was well tolerated, with no alteration of hamster’s weight gain (Supplementary Figure 3). We observed that UDCA reached high proportions of total BA pool only when administered with antibiotics (clindamycin and gentamicin, as described in Materials and Methods), with a 43.5% UDCA peak at D5 (group A) (Figure 3). This high rate of UDCA decreased at 20.56% at D7, two days after stopping antibiotics. Interestingly, without antibiotics, we didn’t observe any similar UDCA peak in feces of hamsters that received UDCA only (max. 4.28% of UDCA at D7). Over the experiment, we noticed that both UDCA and CA concentrations in group A (antibiotics + UDCA) had similar kinetics, evolving in a 1:1 ratio during the first eight days, until UDCA discontinuation (Supplementary Figure 4).

#### Measurement of BA in the Gut, the Entero-Hepatic and the Systemic Circulations

**FIGURE 4:**
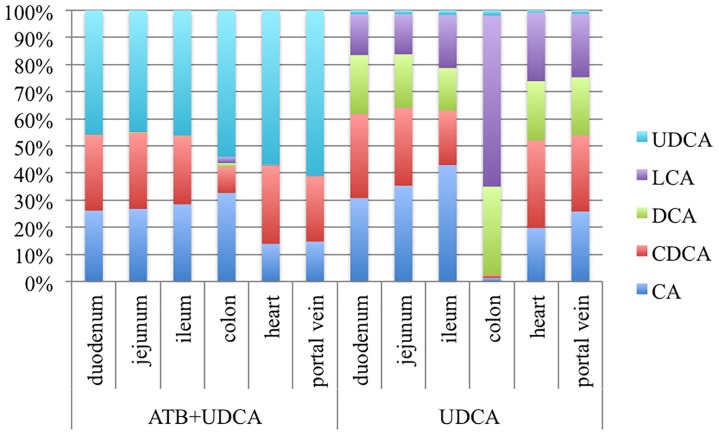
Percentages of bile acids in the different digestive segments, in heart blood and in portal vein blood, in two groups of 3 hamsters each: antibiotics (ATB) + ursodeoxycholic acid (UDCA) and UDCA alone. LCA, Lithocholic acid; DCA, Deoxycholic acid; CDCA, Chenodeoxycholic acid; CA, Cholic acid.

In order to explain this difference between UDCA rates in fecal BA with and without antibiotics, we repeated the pharmacokinetics experiment, adding a sacrifice at D5 in order to determine BA composition in the different intestinal segments and in the systemic and entero-hepatic circulations. Surprisingly, UDCA was found in very small amount in the group that received UDCA alone, in the digestive tract but also in the entero-hepatic circulation (Figure 4). In contrast, LCA was notably increased in this group, compared to the group with antibiotics (+60.8% ± 1.6% in the colon).

#### Infectious Challenge – High Percentages of UDCA in Fecal BA Pool Did Not Prevent CDI

**FIGURE 5:**
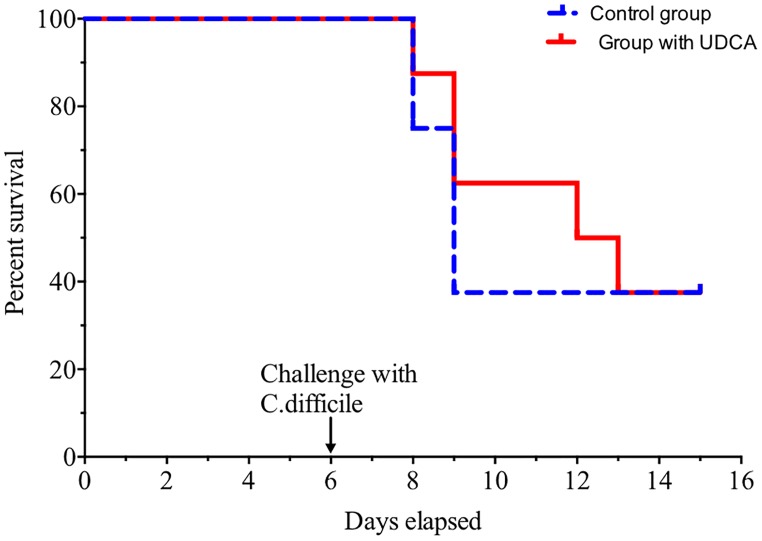
Hamsters’ survival when challenged with spores of *C. difficile* VPI 10463. Solid red line = hamsters treated with ursodeoxycholic acid (UDCA), dotted blue line = control group. Challenge was made at D6, after pre-treatment of hamsters by clindamycin and gentamicin. Difference between groups was not significant (*P* = 0.78, log rank test).

**FIGURE 6:**
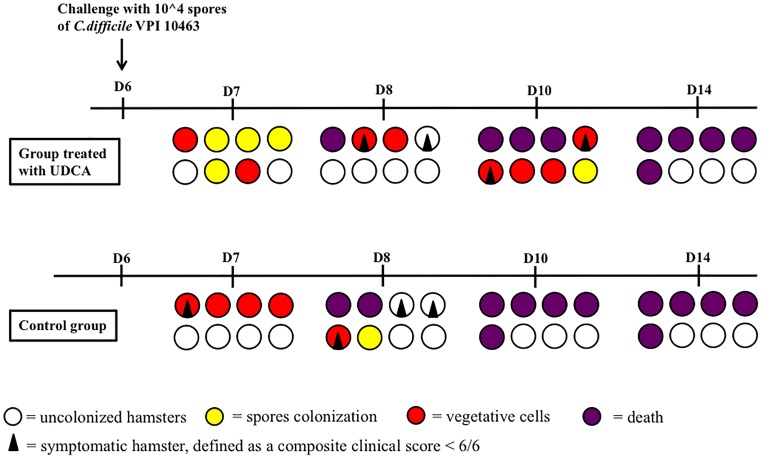
Colonization and death of hamsters challenged with spores of VPI 10463 after pre-treatment with clindamycin and gentamicin. Each circle or square represents the same animal on different days (D) of observation. UDCA, ursodeoxycholic acid.

Overall, five out of eight hamsters died of CDI in the control group and in the UDCA treated group (Figure 5). The delay in mortality observed in UDCA-treated hamsters was not significant. (*P* = 0.78) Figure 6 represents symptoms, colonization by *C. difficile* and death in both groups. We observed that, in the control group, surviving hamsters had never been colonized by vegetative forms of *C. difficile*. On the opposite, in UDCA-treated group, two hamsters were colonized by vegetative forms of *C. difficile* and yet survived. Analysis of fecal BA at D6 (day of *C. difficile* spore gavage) showed high rates of UDCA in hamsters of group A (UDCA + ATB): 44.1% ± 4.0%. UDCA percentage in group A was high and similar in dead and surviving hamsters (Table 1). Similarly to our pharmacokinetics experiment, UDCA percentages dropped to 25% after stopping antibiotherapy for two days, with the same kinetics in dead and surviving hamsters (Supplementary Figure 5). Importantly, in both groups, we observed that dead hamsters had a higher ratio of primary over secondary BA compared to surviving hamsters, with higher concentrations of CA and lower concentrations of LCA (Table 1). The first hamsters to die had the highest ratio of primary on secondary BA (Table 2). CA and CDCA decreased quickly when antibiotherapy was stopped, with a return to baseline of LCA and DCA, indicating a rapid resilience of microbiota transforming primary into secondary BA (Supplementary Figures 6, 7).

**Table 1:** Fecal bile acids percentages in hamsters treated with UDCA versus control group, the day of *C. difficile* challenge.

	Group A (UDCA treated)			Group B (control group)		
	Surviving hamsters	Dead Hamsters	*P*	Surviving hamsters	Dead Hamsters	*P*
CA	21.6 ± 0.9	35.5 ± 0.9	<0.0001	31.7 ± 7.4	64.3 ± 11.5	< 0.0001
CDCA	4.1 ± 0.9	5.0 ± 0.7	0.81	5.5 ± 1.0	8.1 ± 1.0	0.72
DCA	11.9 ± 1.6	9.1 ± 3.2	0.42	39.8 ± 4.2	19.3 ± 9.8	0.0039
LCA	20.8 ± 9.7	3.9 ± 2.0	<0.0001	22.1 ± 3.8	5.7 ± 3.4	0.02
UDCA	40.8 ± 9.8	46.0 ± 3.8	0.14	0.9 ± 0.6	1.6 ± 0.9	0.92
Primary/Secondary BA	1.3 ± 0.7	7.3 ± 3.1	0.086	0.7 ± 0.3	24.6 ± 17.9	0.0007

*Data represent mean ± standard error of the mean. UDCA, ursodeoxycholic acid; CA, cholic acid; CDCA, chenodeoxycholic acid; DCA, deoxycholic acid; LCA, lithocholic acid. Primary/Secondary BA was calculated as the average of each hamster primary (CA + CDCA)/secondary BA (DCA + LCA) ratios (available in Supplementary Table 1).*

**Table 2:** Ratio of primary on secondary fecal bile acids the day of *C. difficile* challenge, per hamster, with their day of death.

Hamster	Day of death	Primary/Secondary bile acids	Hamster	Day of death	Primary/Secondary bile acids
A1	8	16.7	B7	8	95.3
A4	9	12.7	B1	8	17.2
A5	9	2.1	B8	9	9.0
A3	12	3.8	B3	9	1.0
A2	13	1.2	B6	9	0.6
A6		0.5	B2		0.4
A7		0.6	B4		0.4
A8		2.7	B5		1.2

*Primary on secondary ratio was calculated as the average of each hamster primary (CA + CDCA)/secondary BA (DCA + LCA) ratios.*

#### PSC-IBD Patients Treated by UDCA Did Not Present Less CDI Than IBD Patients

We observed that 9.2% (*n* = 41/445) of patients with IBD without PSC (not treated with UDCA) had a CDI at the time of admission for an IBD flare, between September 2012 and May 2014 (Table 3). Unexpectedly, 25% (*n* = 4/16) of patients with PSC-IBD treated with UDCA had a CDI at the time of admission for a flare.

**Table 3:** Number of patients with *C. difficile* infection (CDI), hospitalized for an inflammatory bowel disease (IBD) flare, treated with ursodeoxycholic acid (UDCA) for a primary sclerosing cholangitis (PSC) or without PSC thus untreated.

	CDI	No CDI	% CDI
IBD + PSC treated with UDCA	4	12	25%
IBD without PSC	41	404	9.2%

## Discussion

We confirmed a strong inhibitor effect of UDCA on growth and germination *in vitro*, at 0.05 and 0.1%, competing with 0.1% TCA (with 0.1%: 0.05% ± 0.05% colony forming unit versus 100% ± 0%, *P* < 0.0001). In hamsters, UDCA reached high levels only when administered with antibiotics (43.5% UDCA at D5). Without antibiotics, UDCA was in small amount in feces (max. 4.28%), probably because of UDCA transformation into LCA by gut microbiota. However, UDCA did not protect from CDI *in vivo*: during infectious challenge, mortality was similar in animals treated with UDCA and controls (62.5%, *n* = 5/8, *P* = 0.78). UDCA percentage was high, similar and with the same kinetics in dead and surviving hamsters. However, dead hamsters had a higher ratio of primary over secondary BA compared to surviving hamsters. Finally, in a population at risk (IBD patients), patients treated with UDCA were not protected from CDI: 25% (*n* = 4/12) CDI in PSC-IBD patients versus 9% (*n* = 41/404) CDI in IBD patients.

*In vitro*, germination and growth were inhibited by UDCA 0.05 and 0.1%. In previous studies, UDCA inhibited germination of several strains of *C. difficile* at 2 mM (equal to 0.06%) (Sorg and Sonenshein, 2010). *C. difficile* VPI10463 germination was also inhibited by UDCA, at 0.5, 1, and 2 mM (equal to 0.02, 0.04, and 0.08%), whereas growth of this strain was inhibited by 2 mM (Weingarden et al., 2015). In concordance with our results, lower doses of UDCA (0.0001, 0.001, 0.01%) did not inhibit germination or growth (Theriot et al., 2016). In hamsters, when co-administrated with antibiotherapy, UDCA levels reached 45% of fecal BA, but only 4.28% when administrated alone. We did not find UDCA in the entero-hepatic circulation: ileal reabsorption could not explain the large difference in UDCA fecal concentrations between groups. However, as percentages of LCA were higher in hamsters free of antibiotics, we hypothesized that UDCA was mainly transformed by the microbiota into LCA, as repeatedly described (Fedorowski et al., 1979; Bazzoli et al., 1982; Crosignani et al., 1991). By altering microbiota, antibiotics may inhibit UDCA transformation into LCA, explaining our high levels of UDCA. Interestingly, hamsters treated by antibiotics and UDCA had a fecal ratio of CA/UDCA close to 1:1, compatible with the inhibitory effect of UDCA on germination and growth of *C. difficile* observed *in vitro*. However, UDCA at 50 mg/kg/day failed to prevent CDI or to reduce colonization or mortality. Dead hamsters had a higher ratio of primary to secondary BA than surviving hamsters at the time of *C. difficile* challenge, supporting the hypothesis of an unbalance of primary and secondary BA leading to CDI. Increasing UDCA in the fecal BA pool did not change this ratio. However, two surviving hamsters treated with UDCA+antibiotics were colonized with vegetative forms of *C. difficile*, but then eliminated the bacteria without developing an infection. These vegetative forms could have been neutralized by UDCA. These results differ from a study that successfully tested UDCA *in vivo* on a single patient with CDI pouchitis, refractory to antibiotherapies and fecal microbiota transplantation (Weingarden et al., 2015). However, this patient had a total colectomy for ulcerative colitis, which limits UDCA microbial transformation. Recently, a study described the off-label use of UDCA as salvage therapy to prevent CDI recurrence in 16 patients with contraindications for fecal microbiota transplant, with 87.5% remaining free of recurrent CDI at median follow-up of 264 days from UDCA prescription (Webb et al., 2018). This was a small case series in a highly selected population with no control group, leading to limited interpretation. However, it raises the question of the validity of the animal model. IBD patients are known to present lower secondary BA in feces, worsened by the activity of the disease (Jones et al., 2013), with BA profiles spontaneously close to what is observed after antibiotics administration. They also present an increased risk of CDI. We observed from a cohort that IBD patients treated for PSC with UDCA presented more CDI at entrance at the hospital for an IBD flare than patients with IBD alone: 25% versus 9%, respectively. These results are in concordance with the inefficiency of UDCA found in the hamster model, even if there are some confounding factors. Indeed, patients with PSC have elevated concentrations of taurine and glycine conjugates of primary BA, including CA, which triggers germination and growth of *C. difficile* (Trottier et al., 2012).

Our *in vivo* study presents some limitations. First, we did not use empty capsules for control groups. However, the capsules we used were made of 100% porcine gelatin, one of the most common components of traditional pharmacopeia, considered to have no interactions with drugs and metabolism. Second, we did not test higher concentrations of UDCA than 50 mg/kg/day. We chose usual – or slightly superior – doses, because UDCA has a marketing authorization for cholestatic liver diseases at 13–30 mg/kg/day and the trial that tested higher doses was prematurely stopped for serious adverse events (Lindor et al., 2009). An increased risk of colorectal cancer was also found with higher doses of UDCA for PSC (Eaton et al., 2011). Concerning the animal model, we chose hamster over mouse, first, because hamsters have very little muricholic BA. Muricholic BA, highly present in mice, are strong inhibitors of *C. difficile* germination and growth (Francis et al., 2013b). Thus, hamsters are very sensitive to *C. difficile* and are commonly used for testing new treatments against *C. difficile*, but it may have been a too sensitive model. Furthermore, one could think that UDCA inefficiency was related to the use of the strain VPI10463. There is indeed a possible correspondence of variation in germination responses across isolates with mutations in CspC, the *C. difficile* receptor of BA (Bhattacharjee et al., 2016) and strains 630Δerm and R20291 seemed even more susceptible to UDCA than strain VPI10463. However, our *in vitro* results with VPI10463 were satisfying, with 100% inhibition of CFU from spores with UDCA 0.1%, and this strain is currently used in the hamster model (Hutton et al., 2014; Secore et al., 2017). Also, we could underline that the quick decrease of UDCA in fecal BA could have impaired our results. However, surviving and dead hamsters had the same kinetics of UDCA fecal percentages decrease. Finally, the discordance between *in vitro* and *in vivo* could be explained by a different bioavailability of UDCA, quite lipophilic, in colon. In addition, other steroids present in the colon may also play an undetermined role in germination and growth of *C. difficile*: ileum and colon content are a complex medium and BA may not be the only actors to facilitate CDI. We systematically analyzed a wide BA spectrum, in order to rule out any confounding BA triggering germination or growth. It is the first time that UDCA, whose efficiency had been proved *in vitro*, was tested at usual doses *in vivo* during an infectious experiment. It is interesting to note that in our experimental conditions, at UDCA doses close to clinical practice, UDCA could not prevent CDI, even at high levels in fecal BA. Finally, the observation on PCS-IBD patients under UDCA-therapy is based on a limited number of patients with PSC and without matching; furthermore patients with a PSC-IBD have a less active disease (Sokol et al., 2008) and the control of IBD limits CDI, on the contrary of what was observed here. Despite these limits, in this population at risk for CDI, long-term treatment by UDCA did not prevent infection.

Our results in animal and humans suggest that UDCA, at usual doses, is inefficient to prevent CDI. Among other options, working with higher doses of UDCA must be considered with caution, given the toxicity of UDCA metabolites, especially LCA.

## Data Availability

The raw data supporting the conclusions of this manuscript will be made available by the authors, without undue reservation, to any qualified researcher.

## Author Contributions

L-JP, DR, BC, CJ, and HD contributed conception and design of the study. L-JP, DR, HS, LB, LH, SH, CJ, and HD contributed to the acquisition of data, analysis and interpretation of data. L-JP and HD performed the statistical analysis. L-JP wrote the first draft of the manuscript. HD and CJ wrote sections of the manuscript. MD, TE, and AB made critical revision of the manuscript for important intellectual content. All authors contributed to the revision of the manuscript, read and approved the submitted version.

## Conflict of Interest Statement

HD has worked as a scientific advisor for Ipsen laboratory and Biocodex laboratory. HS has worked as a consultant for Enterome, Maat, and is the co-founder of Nextbiotix. The remaining authors declare that the research was conducted in the absence of any commercial or financial relationships that could be construed as a potential conflict of interest.

## Abbreviations

BA: Bile Acids
BHIS: Brain Heart Infusion Supplemented
CA: Cholic Acid
CDCA: Chenodeoxycholic acid
CDI: *Clostridium difficile* infection
CFU: Colony forming unit
DCA: Deoxycholic acid
IBD: Inflammatory bowel disease
LCA: Litocholic acid
OD_600_: Optical density at 600 nm
PSC: Primary sclerosing cholangitis
TCA: Taurocholic acid
UDCA: Ursodeoxycholic acid.
