# Supplementary material for: Inhibitory Effect of Ursodeoxycholic Acid on Clostridium difficile Germination Is Insufficient to Prevent Colitis: A Study in Hamsters and Humans
Source: Front Microbiol. 2018 Nov 22;9:2849. doi: 10.3389/fmicb.2018.02849 (PMC6262072; doi:10.3389/fmicb.2018.02849)
Supplement: Supplementary file 1 [file Table_1.DOCX]

Supplementary Material

**Inhibitory effect of ursodeoxycholic acid on *Clostridium difficile* germination is insufficient to prevent colitis: a study in hamsters and humans**

Lola-Jade Palmieri, Dominique Rainteau, Harry Sokol, Laurent Beaugerie, Marie Dior, Benoit Coffin, Lydie Humbert, Thibaut Eguether, André Bado, Sandra Hoys, Claire Janoir, Henri Duboc*

*** Correspondence:** Henri Duboc, henri.duboc@aphp.fr

# Supplementary Figures and Tables

## Supplementary Figures

**Supplementary Figure 1: *C. difficile* VPI 10463 (A), 630** **Δ*erm* (B), R20291 (C) growth curves** with 0.1% ursodesoxycholic acid (UDCA) (purple), 0.05% UDCA (green), 0.01% UDCA (red) and in control group with ethanol (2% ethanol [EtOH]) (blue). According to the strains, significance was achieved at 24h with 0.05% and 0.1% UDCA, compared to the control (Two-way ANOVA, ***=p<0.001, ****=p<0.0001).

**Supplementary Figure 2: Germination curves of *C. difficile* 630Δ*erm* spores** (ratios of the optical densities [OD] at various time points to the starting optical density are plotted against time).

**Supplementary Figure 3: Hamsters weight curve during pharmacokinetics experiment**, in the three groups (antibiotics [ATB] + ursodesoxycholic acid [UDCA], antibiotics and UDCA alone). No significant difference between groups was observed (two-way ANOVA).

**Supplementary Figure 4: Fecal bile acids percentages in time.** Group A (**A**) received ursodesoxycholic acid (UDCA) with antibiotics between D+1 and D+6, Group B (**B**) received only antibiotics according to the same modalities and Group C (**C**) received only UDCA according to the same modalities. CA = Cholic acid.

**Supplementary Figure 5:** Percentage of UDCA in fecal bile acid pool over time in hamsters treated with UDCA, that survived (green curves), or that died (red curves). Each curve represents a single animal. UDCA= ursodesoxycholic acid

**Supplementary Figure 6:** Evolution of fecal bile acids percentages over time, in group A (treated with ursodeoxycholic acid [UDCA]). Hamsters received antibiotics between D1 and D5, UDCA between D1 and D12. They were challenged with *C. difficile* on D6. Data is represented with mean and standard error of the mean. CA: cholic acid, CDCA: chenodeoxycholic acid, DCA: deoxycholic acid, LCA: lithocholic acid

**Supplementary Figure 7:** Evolution of fecal bile acids percentages over time, in group B (control group). Hamsters received antibiotics between D1 and D5. They were challenged with *C.difficile* on D6. Data is represented with mean and standard error of the mean. CA: cholic acid, CDCA: chenodeoxycholic acid, DCA: deoxycholic acid, LCA: lithocholic acid

## Supplementary Tables

**Supplementary Table 1:** List of the 28 bile acids species measured in feces

| **Primary bile acids** | **Secondary bile acids** | **UDCA** |
| --- | --- | --- |
| CA | DCA | UDCA |
| TCA | TDCA | TUDCA |
| GCA | GDCA | GUDCA |
| CA-3S | DCA-3S | UDCA-3S |
| CDCA | LCA | TUDCA-3S |
| TCDCA | TLCA | GUDCA-3S |
| GCDCA | GLCA |  |
| CDCA-3S | LCA-3S |  |
| HCA | TLCA-3S |  |
| MCA | GLCA-3S |  |
|  | HDCA |  |
|  | THDCA |  |
|  |  |  |
| CA: Cholic acid | | |
| CDCA: Chenodeoxycholic acid | | |
| DCA: Deoxycholic acid | | |
| LCA: Lithocholic acid | | |
| UDCA: Ursodeoxycholic acid | | |
| HCA: Hyocholic acid | | |
| HDCA: Hyodeoxycholic acid | | |
| MCA: Muricholic acid | | |
| T and G prefix indicates Tauro or Glyco conjugation | | |
| 3S suffix indicates Sulfation on carbon 3 | | |
